# Supplementary material for: Quantized charge fractionalization at quantum Hall Y junctions in the disorder dominated regime
Source: Nat Commun. 2021 Jan 7;12:131. doi: 10.1038/s41467-020-20395-7 (PMC7788083; doi:10.1038/s41467-020-20395-7)
Supplement: Supplementary file 1 — Supplementary Information [file 41467_2020_20395_MOESM1_ESM.pdf]

Supplementary information for
‘Quantized charge fractionalization at quantum Hall Y junctions in the disorder dominated regime’

Chaojing Lin[1,2], Masayuki Hashisaka[3], Takafumi Akiho[3], Koji Muraki[3], and Toshimasa Fujisawa[1]

[1]Department of Physics, Tokyo Institute of Technology, 2-12-1-H81 Ookayama, Meguro, Tokyo 152-8551, Japan,

[2]Tokyo Tech Academy for Super Smart Society, Tokyo Institute of Technology, 2-12-1 Ookayama, Meguro, Tokyo 152-8551, Japan.

[3]NTT Basic Research Laboratories, NTT Corporation, 3-1 Morinosato-Wakamiya, Atsugi, Kanagawa 243-0198, Japan.

Supplementary Note 1: Sample and low-frequency transport characteristics

Supplementary Fig. 1a shows an optical micrograph of the chip that we investigated. The upper and lower parts of the chip were used as devices #1 and #2, respectively. The AlGaAs/GaAs heterostructure with a two-dimensional electron gas (2DEG) was partially removed by chemical etching (dark regions). Whereas several ohmic contacts Ω_I, Ω_{II}, … were formed on the outer edge of the 2DEG, a few ohmic contacts Ω_1, Ω_1’, Ω_2, Ω_2’, … were formed on the inner edges to provide the Corbino geometry. The bulk filling factor ν_B in the ungated region was set by applying a perpendicular magnetic field B. By applying gate voltage V_g to large gates G_1 and G_2, the filling factor ν_G under the gated regions was selected. Small gates G_I, G_I’, G_D, and G_D’ were used as charge injectors and detectors for devices #1 and #2.

Four-terminal dc conductance measurements were performed using the setup shown in Supplementary Fig. 1a. With source voltage V_s = 30 μV (37 Hz) applied to ohmic contact Ω_3, the two-terminal conductance G (= I/V_s) was obtained by measuring the current I at Ω_4. In addition, the longitudinal voltage V_{xx} was measured using Ω_1 and Ω_2. Supplementary Fig. 1b shows a color plot of the measured V_{xx} as a function of gate voltage V_g and magnetic field B under dark conditions. The overall patterns in V_{xx} can be understood with the variation in ν_B in the bulk shown by horizontal lines and ν_G under the gate shown by inclined lines. Vanishing V_{xx} regions (white regions) show negligible bulk scattering in both the gated and ungated QH states. We assumed the same QH states were formed under gate G_1 in device #1.

For example, a system with $\nu_B = 1$ and $\nu_G = 2/3$ was prepared at V_g = -0.08 V and B = 7.5 T. In this case, a complex $\Delta\nu = 2/3$ channel made of counterpropagating $\Delta\nu = 1/3$ and 1 channels was formed by edge reconstruction with a non-monotonic variation of ν from 0 through 1 to 2/3 [1]. Consequently, the single $\Delta\nu = 1/3$ channel yielded a closed loop along the side of gate G_2, as shown by the red line in Supplementary Fig. 1a. This channel is coupled to four $\Delta\nu = 1$ channels (blue) connected to ohmic contacts via the complex $\Delta\nu = 2/3$ channels (parallel blue and red lines). Transport is allowed by

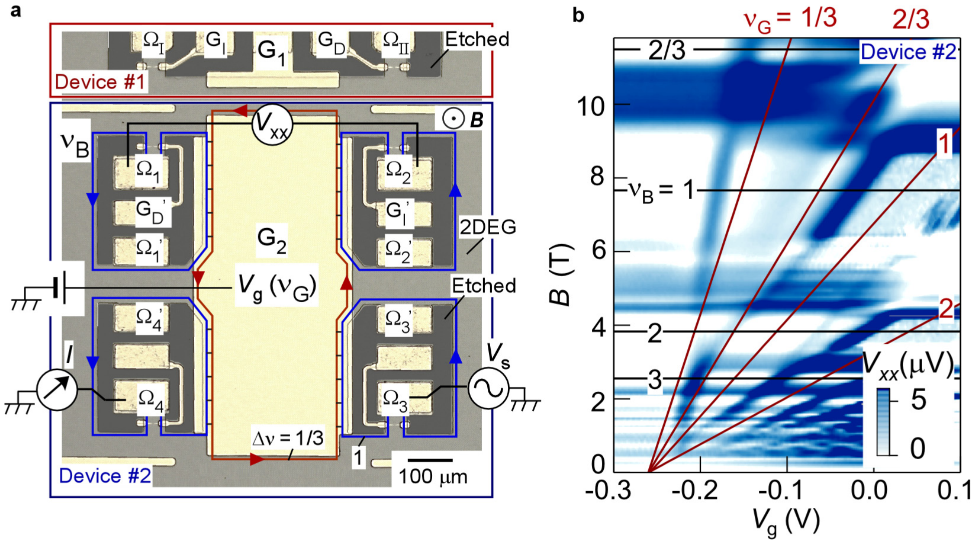

Supplementary Figure 1. Low-frequency characteristics of the QH states. a, Optical micrograph of devices #1 and #2 with measurement setup for four-terminal dc measurements. **b**, Color plot of V_{xx} as a function of gate voltage V_g and magnetic field B. The QH states are labeled ν_B for the bulk (horizontal lines) and ν_G under the gate (inclined lines).

scattering in the $\Delta\nu$ = 2/3 channels. We find that the two-terminal conductance $G \cong 1/3\ e^2/h$ (not shown) agrees well with the full equilibration. The length of the complex $\Delta\nu$ = 2/3 channel, ~ 300 μm, is longer than typical equilibration length, ~ 10 μm obtained in our previous study for a similar 2DEG wafer [2].

Supplementary Note 2: Charge waveforms obtained from device #1

Supplementary Fig. 2a shows the current I_D as a function of delay time t_d in a wide range of V_g from -0.3 V to +0.3 V, obtained at ν_B = 2/3 (B = 11.5 T) in device #1. A single wave packet was observed at V_g ~ -0.27 V (ν_G = 0), where the packet propagated along the composite $\Delta\nu$ = 2/3 channel comprising $\Delta\nu$ = 1 (blue) and 1/3 (red) channels along the perimeter of the gate, and V_g = +0.03V (ν_G = 2/3), where the packets propagated along the etching step under the gate, as shown in the insets. The former indicates a faster velocity of ~ 70 km/s (short time-of-flight for a long distance) than the latter (~ 20 km/s). This can be understood based on the screening effect of the metal gate [3, 4].

When V_g was increased above +0.18 V (ν_G = 1), two distinct packets appeared in the I_D profile. They are associated with multiple fractionalizations at the Y_C and Y_N junctions, as illustrated in the inset. The quantized fractionalization of factor 1/3 suggests the generation of multiple wave packets (2q/3, 2q/9, …) toward the detector. This was experimentally confirmed as described in the main paper (see Fig. 3b). The red highlighted traces at V_g = -0.3 V and V_g = +0.26 V are shown as traces (i) and (ii), respectively, in Fig. 2b of the main paper.

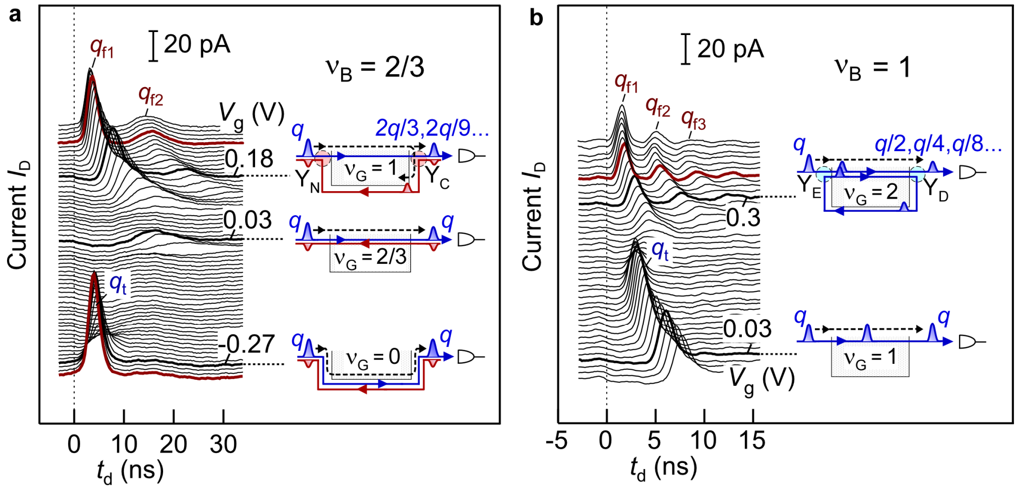

Supplementary Figure 2. Charge waveforms in device #1. a, Waterfall plot of current I_D as a function of delay time t_d for various V_g values from -0.3 V (the bottom trace at $\nu_G = 0$) to +0.3 V (the top trace at $\nu_G \cong 1$) with step 0.01 V obtained at B = 11.5 T ($\nu_B = 2/3$). **b**, Current I_D traces for various V_g values from 0 V (the bottom trace at $\nu_G = 1$) to +0.4 V (the top trace at $\nu_G \cong 2$) with step 0.01 V obtained at B = 7.5 T ($\nu_B = 1$). The red highlighted traces are presented in Figs. 2b and 2c of the main paper.

The data for quantized fractionalization in the integer QH regime are shown in Supplementary Fig. 2b with I_D profiles measured in a wide range of V_g from 0 V ($\nu_G \sim 1$) to +0.4 V ($\nu_G \sim 2$) at $\nu_B = 1$ (B = 7.5 T). A single wave packet was observed at approximately $\nu_G = 1$, where a single $\Delta\nu = 1$ channel was formed under the gate, as shown in the inset. By contrast, multiple peaks appeared at approximately $\nu_G = 2$. This can be understood by the multiple fractionalizations between junctions Y_E and Y_D, as illustrated in the inset. The red highlighted trace at V_g = +0.34 V is shown in Fig. 2c of the main paper.

Supplementary Note 3: Waveforms obtained from device #2

As described in the main paper, an extremely slow propagation was observed for the $\Delta\nu = 1/3$ interface channel with $\nu_G = 2/3$ in the gated region. Because the wave packet was broadened significantly, it was difficult to identify multiple peaks in the measurement using device #1. Hence, we used the setup shown in Supplementary Fig. 3a, where $\nu_G = 2/3$ and $\nu_B \cong 1$ states were formed in the gated and bulk regions, respectively, at B = 9.5 T. Charge q generated with gate G_I' experiences fractionalizations first at junction Y_C and then at Y_N before reaching the detector gate G_D'. In this study, we focused on the fractionalized charge $q/3$ travelling in the interface channel $\Delta\nu = 1/3$ (red line) formed between the $\nu_G = 2/3$ and $\nu_B \cong 1$ regions. The other charge $2q/3$ fractionalized at Y_C was absorbed in the grounded ohmic contact and therefore did not affect the measurement. This enables us to focus on the transport in the $\Delta\nu = 1/3$ channel.

Trace (i) in Supplementary Fig. 3b shows the reference waveform obtained with $\nu_G = 0$, where a single $\Delta\nu = 1$ channel was formed between the injector and detector. Trace (ii) shows the wave packet obtained with $\nu_G = 2/3$ for studying fractionalization, as illustrated in the inset. The fractionalized wave packet in trace (ii) is significantly delayed and broadened as compared with trace (ii) in Fig. 2b of the main paper. To obtain a reasonable signal-to-noise ratio, we set the width of the detector pulse, t_w = 260 ns, which was comparable with the width of this fractionalized wave packet. This large t_w was directly reflected in the width of the reference wave packet in (i); otherwise, a much narrower

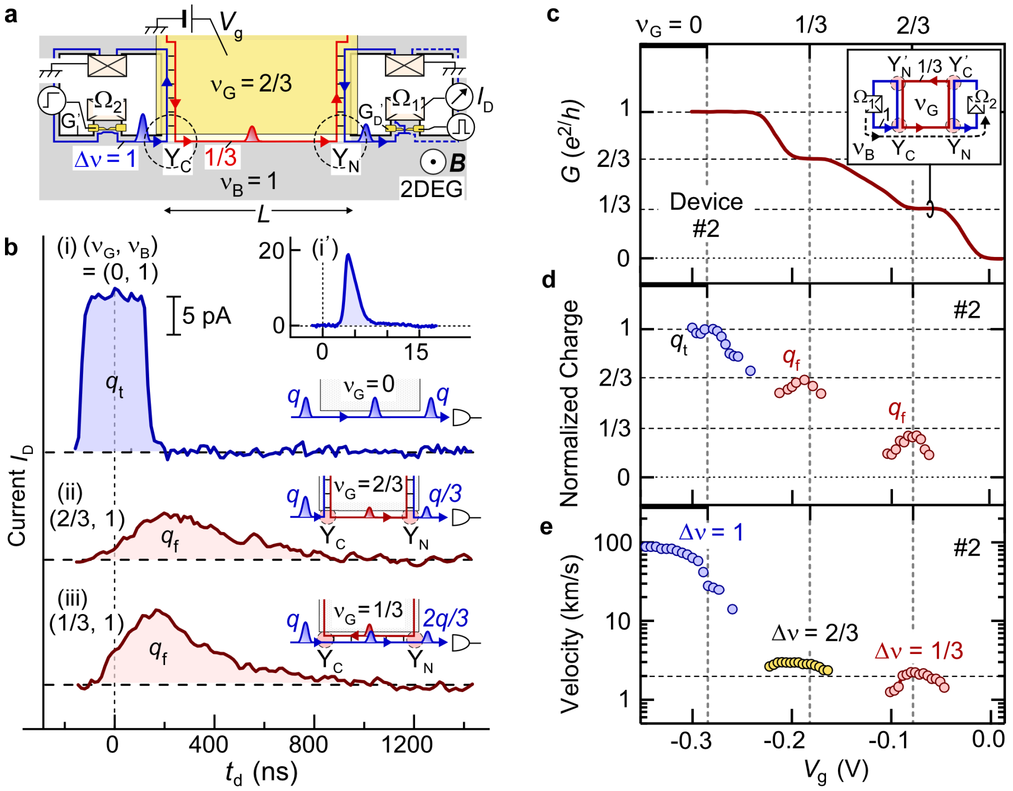

Supplementary Figure 3. Fractionalization of charge wave packets. a, Measurement setup for Corbino geometry in device #2. A wide QH region (L = 420 μm) and junctions Y_N and Y_C were formed at $\nu_G = 2/3$ and $\nu_B = 1$. The initial wave packet was excited by applying a voltage step (V_I = 15 mV) to gate G_I', and fractionalized wave packets were detected by applying a voltage pulse (V_D = 20 mV) to gate G_D'. **b**, Typical charge waveforms obtained in current traces $I_D(t_d)$; reference trace (i) at $\nu_G = 0$ (V_g = -0.3 V), trace (ii) for fractionalized packet at $\nu_G = 2/3$ (V_g = -0.08 V), and trace (iii) at $\nu_G = 1/3$ (V_g = -0.18 V) under $\nu_B \cong 1$ (B = 9.5 T) and t_w = 260 ns. The trace (i') in the inset shows a reference trace obtained with t_w = 0.08 ns. Propagation of charge wave packets are illustrated in the insets. **c-e**, V_g-dependence of two-terminal conductance G in **c**, charges q_t and q_f normalized by q_t (≅ 8000e) at V_g = -0.3 V in **d**, and charge velocities for $\Delta\nu$ = 1/3, 2/3, and 1 channels in **e**. The corresponding ν_G is shown on the top scale. The inset to **c** shows charge fractionalizations under $\nu_G = 2/3$ and $\nu_B = 1$.

wave packet was observed for t_w = 0.08 ns, as shown for trace (i') in the inset. We evaluated the reference charges q_t and fractionalized charge q_f with the same t_w, as shown in Supplementary Fig. 3d. The normalized charge q_f/q_t approached 1/3 at ν_G = 2/3. Hence, the fractionalization ratio remains unchanged even when the wave packet is significantly delayed and distorted, as summarized in Fig. 4c of the main paper.

It should be noted that a clear wave packet was observed in trace (iii) of Supplementary Fig. 3b taken at ν_G = 1/3, where a different type of complex $\Delta\nu$ = 2/3 channel was formed between the ν_G = 1/3 and ν_B = 1 regions, as shown in the inset. The normalized charge for this packet was approximately 2/3, as shown in Supplementary Fig. 3d. Furthermore, the data set above supports fractionalization factor 1/3 and charge conservation in the system.

Moreover, the quantized fractionalization is consistent with the dc conductance measurement shown in Supplementary Fig. 3c. The two-terminal conductance between ohmic contacts Ω_1 and Ω_2 with other ohmic contacts floating is plotted as a function of V_g. The clear plateau of $G = e^2/3h$ at ν_G = 2/3 (V_g = -0.08 V) indicates full equilibration in the complex $\Delta\nu$ = 2/3 channels. This can be understood by the 1/3 charge fractionalization at junction Y_C in path Ω_1 - Y_C -Y_N - Ω_2, as shown in the inset.

Supplementary Fig. 3e shows the velocity estimated from the time-of-flight. Whereas the velocity of the edge channel $\Delta\nu$ = 1 between ν_G = 0 and ν_B = 1 was comparable to those in previous reports [4-6], the velocity of the $\Delta\nu$ = 1/3 interface channel between the ν = 1 and 2/3 regions was particularly slow at ~2 km/s (at B = 9.5 T), suggesting a large geometric capacitance between the channel and the gate, as discussed in the main paper.

References

[1]. MacDonald, A. H. Edge states in the fractional-quantum-Hall-effect regime. *Phys. Rev. Lett.* **64**, 220 (1990).

[2]. Lin, C. J., Eguchi, R., Hashisaka, M., Akiho, T., Muraki, K. & Fujisawa, T. Charge equilibration in integer and fractional quantum Hall edge channels in a generalized Hall-bar device. *Phys. Rev. B* **99**, 195304 (2019).

[3]. Ernst, G., Zhitenev, N. B., Haug, R. J. & Klitzing, K. von. Dynamic excitations of fractional quantum Hall edge channels. *Phys. Rev. Lett.* **79**, 3748 (1997).

[4]. Ashoori, R. C., Stormer, H. L., Pfeiffer, L. N., Baldwin, K. W., West, K. Edge magnetoplasmons in the time domain. *Phys. Rev. B* **45**, 3894 (1992).

[5]. Kamata, H., Ota, T., Muraki, K. & Fujisawa, T. Voltage-controlled group velocity of edge magnetoplasmon in the quantum Hall regime. *Phys. Rev. B*. **81**, 085329 (2010).

[6]. Kumada, N., Kamata, H. & Fujisawa, T. Edge magnetoplasmon transport in gated and ungated quantum Hall systems. *Phys. Rev. B* **84**, 045314 (2011).
